# Splenogonadal fusion: Report of four cases and review of the literature

**DOI:** 10.3892/etm.2013.1207

**Published:** 2013-07-05

**Authors:** WAN-FU LI, MEI-XIANG LUAN, ZHU MA, YA-JUN CHEN

**Affiliations:** 1 Department of Pediatric Surgery, The First Affiliated Hospital of Xinjiang Medical University, Urumqi, Xinjiang 830054, P.R. China; 2 Department of General Surgery, Beijing Children’s Hospital, Capital University of Medical Sciences, Beijing 100045, P.R. China

**Keywords:** splenogonadal fusion, cryptorchidism, spleen, testis, laparoscopy

## Abstract

Splenogonadal fusion (SGF) is a rare congenital abnormality that affects children of both genders. Very few cases of SGF have been diagnosed preoperatively. In this study, the surgical findings and laparoscopic treatment of four children with SGF associated with intra-abdominal cryptorchidism are described. Laparoscopy was demonstrated to be the only accurate exploratory procedure for the diagnosis and surgical treatment of SGF with non-palpable testis.

## Introduction

Splenogonadal fusion (SGF) is a rare congenital anomaly in which close proximity of spleen and gonad during early embryological development facilitates fusion. SGF may be divided into continuous or discontinuous types. The continuous type of SGF describes the gonad attached to the spleen. The discontinuous type consists of gonadal fusion with an accessory spleen or ectopic splenic tissue. The diagnosis of this uncommon anomaly is rare and difficult to diagnose preoperatively. Certain patients with SGF have undergone unnecessary orchidectomy due to the presence of a testicular lump (1). Therefore, re-evaluation of the diagnosis and surgical treatment of SGF is necessary. In the current study, we report four cases of children with SGF, three of which were treated by laparoscopy. Combined with the relevant literature, we discuss the diagnosis and treatment of SGF, and the value of exploration by laparoscopy. This study was approved by the ethics committee of The First Affiliated Hospital of Xinjiang Medical University. The informed consent was obtained from the family of all patients.

## Case reports

### Case 1

**Figure 1:**
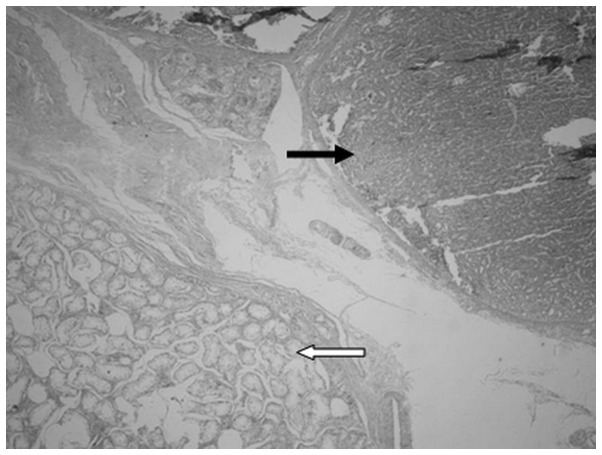
Case 1. Under a light microscope (magnification ×40), spleen tissue (black arrow) and extruded testicular tissue (white arrow) are visible.

A male, 6 years old, presented with a painless mass in the left scrotum. Upon physical examination, a swollen testicle ~2.5×2.5×4.5 cm^3^ in size, of medium hardness without any tenderness was palpable in the left scrotum. Surgery was performed under general anesthesia. During the surgery, a reddish-brown mass attached to the upper pole of the left testis was identified. The mass and testis were free from other intra-abdominal structures. The mass was confined to an intact capsule on the upper pole of the testis and occupied one-third of the volume of the testis. Postoperative examination of the specimen under the microscopic revealed it was spleen tissue that was separated from the surrounding compressed testicular tissue (Fig. 1).

### Case 2

**Figure 2:**
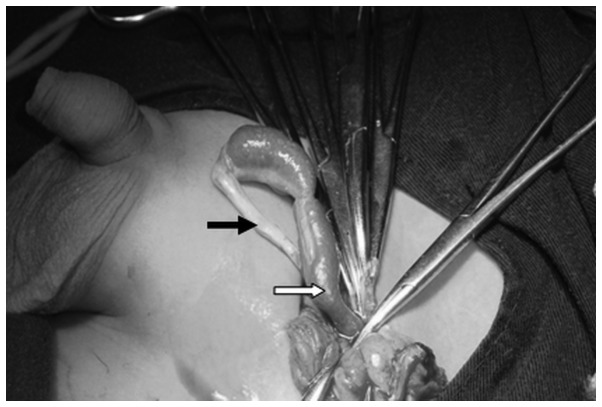
Case 2. Cord-like tissuevisible in the hernia sac; the remote cord-like tissue is thin and connect with the testicle (black arrow) while the proximal streaks are thicker and lead intra-abdominally (white arrow).

**Figure 3:**
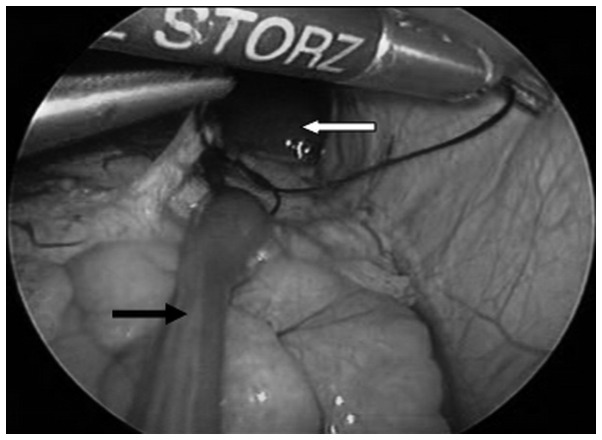
Case 2. During laparoscopy, the streak (black arrow) is observed to issue from the splenic hilum and connect with the spleen (white arrow). The proximal cord-like tissue is ligatured prior to being resectioned.

A male, 7 years old, presented with a left inguinal mass. It was a soft mass with a clear boundary, which was palpable from the left groin area to the scrotum, but was not able to be completely pushed back into the abdominal cavity. During laparoscopy, a congenital hernia was confirmed by the red cord-like tissue in the left inguinal region that connected to the testicle, entered into the left internal inguinal ring and were fused to the hilum of the spleen. The diameter of the cord-like tissue was ~1.0 cm. The proximal cord-like tissue was ligatured prior to being resectioned (Figs. 2 and 3). Postoperative examination of the specimen under the microscope revealed it was spleen tissue.

### Case 3

**Figure 4:**
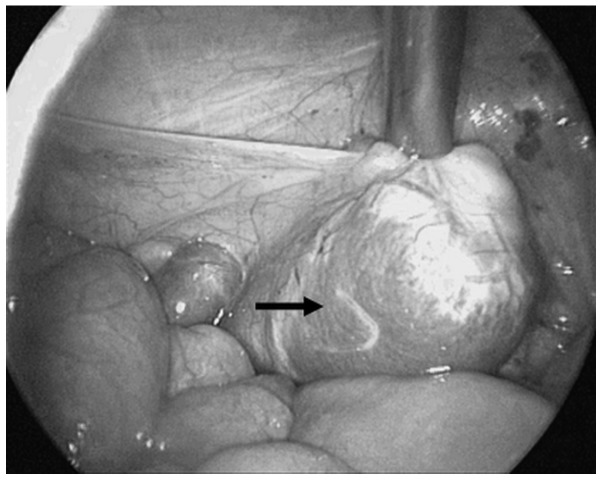
Case 3. Laparoscopy shows that a testis-like mass (black arrow) is present at the inner ring of inguinal canal.

A male, 2-years-old, presented with bilateral undescendent testes. The penis was bent downwards, while the urethra was located at the junction of the penis and scrotum. The testis were not palpable in bilateral scrotums and bilateral inguinal region. Ultrasound demonstrated there was no testicular-like mass in the bilateral inguinal and abdominal cavities. During laparoscopy, a testicle-like mass was located in the left iliac fossa. It was dark red, with clear fixed boundaries and measured 2.0×3.0×4.0 cm^3^ (Fig. 4). The removal of the left testis-like mass was followed by the high ligature of the spermatic vein. The right testicle was pulled down and fixed in a secondary surgery. Pathological examination of the mass revealed SGF.

### Case 4

A male, 12-years-old, presented with bilateral undescendent testes. The penis had a postoperative appearance, a bend in it having been corrected. The urethra was located in the glands. The bilateral scrota were underdeveloped, without the testis. Surgical scars were visible at the bilateral inguinal region, but there was no testis-like mass. An oval-shaped mass shadow on the lower pole of the left kidney was confirmed by MRI. It was ~2.3×4.0 cm^2^ in size with a uniform signal and clear boundary. The location and shape of the spleen was noted to be normal during the laparoscopy. A testis-like reddish brown mass was observed that was attached to the lower pole of the left kidney, with clear, fixed boundaries and measuring 2.0×2.5×4.0 cm^3^. The right testicle was pulled down and fixed, while the left, testis-like mass was removed. Pathological examination of the specimen obtained from the left side revealed SGF.

## Discussion

SGF is a rare congenital anomaly, between the spleen and a gonad or mesonephric derivatives, almost always presenting on the left side in males. The male preponderance may be due to the fact that the male sex gland is located superficially and is easily located. Female gonads are inside the body and have fewer complications than their male counterparts. SGF is often discovered accidentally during gynecological surgery, with a higher prevalence in Caucasian individuals, followed by that in African descent and other ethnicity (2,3). SGF is common in children and adolescents. The number of cases reported in patients <10 years of age account for 50% of the total cases reported, while patients <20 years old account for 70% (1). The four patients in the current study are all male, three of the four patients are <10 years old, and all anomalies occurred on the left side.

SGF may be classified into two types, continuous and discontinuous, but clinically there are no significant differences between the two types (4). Le Roux and Heddle (5) speculated that discontinuous SGF is a rare type of lienculus. Case 2 is an example of continuous SGF, and postoperative examination of the specimen under a microscope revealed that it was spleen tissue. The other three cases are examples of discontinuous SGF in which no cord-like tissue was connected with the spleen.

Cryptorchidism and inguinal hernias are the most common malformations associated with SGF. In total, 31% of SGF patients are diagnosed with cryptorchidism or inguinal hernias, and in 59% of cases the cryptorchidism is diagnosed as bilateral (6). In cases of continuous SGF, ~50% are accompanied by other congenital malformations (7). The incidence rate for continuous SGF is five-fold higher than that of discontinuous SGF and most cases are accompanied by limb defect syndrome (8,9). Bonneau *et al*(10) reviewed 29 cases of splenogonadal fusion limb defect syndrome (SGFLD), of which 24 cases (82.7%) were continuous SGF, while 70% were associated with micrognathia. Approximately one-fifth of continuous SGF cases also have other major congenital defects, such as limb hypoplasia, micrognathia, cardiac defects, palatal defects and anal defects. Karaman and Gonzales (4) reported a case of both transverse testicular ectopia and SGF. In the current study, case 1 has no associated malformation, case 2 has a left inguinal hernia and cases 3 and 4 have bilateral cryptorchidism and hypospadias.

The diagnosis of SGF prior to surgery is challenging. SGF typically presents as an asymptomatic testicular mass and other manifestations may include acute testicular pain and swelling caused by ectopic splenic tissue infections (11), but the actual disease itself lacks characteristic features. In the current study, case 1 was undergoing treatment due to a left scrotal mass, case 2 was undergoing treatment for a left inguinal hernia and cases 3 and 4 were being treated for bilateral cryptorchidism. All four cases were inaccurately diagnosed prior to surgery. The lack of awareness of SGF is a major factor in its misdiagnosis. Imaging methods, including B-type ultrasonography, computed tomography (CT), magnetic resonance imaging(MRI) and ^99^TC^m^ spleen scanning, aid with the diagnosis of SGF (12–15). However, laparoscopies have achieved improved diagnoses and management of SGF (16). In the current study, three of the four cases were treated by laparoscopy, which aided with diagnosis and surgery.

The decision to resection the entire splenogonadal tissue was based upon the anomalous appearance of the testicle and its fusion to ectopic splenic tissue. Once an accurate diagnosis has been achieved without significant complications, particularly in the scrotum, no surgery is required. Even if surgery is performed, in most cases the testis may be preserved. Performing laparoscopy and testicle-sparing surgery is advised. Spleen tissue is easily separated from the gonad and as a result the testis may be retained, unless there is a high degree of cryptorchidism. If a tumor is suspected, frozen sectioning may aid with the diagnosis. Currently, to the best of our knowledge, there have been no reports of malignant SGF.

In conclusion, the diagnostic evaluation of patients with an abnormal gonad is complex due to multifactorial etiopathogenesis and the rarity the condition. The primary aim of diagnosis is to rule out malignancy. We suggest three steps that should be considered when diagnosing and treating SGF. Firstly, a mass found at birth growing slowly for several years in a benign condition should be considered. Secondly, various imaging techniques should be used to investigate the nature of the mass. Thirdly, in doubtful cases, a biopsy should performed during surgery or, preferably prior to making an incision, for example, by needle biopsy, punch biopsy or a classical bivalve biopsy and regional node evaluation. If the mass proves to be malignant, a radical resection should be performed immediately. If the malignancy is unconfirmed, an orchiectomy is sufficient for further pathological study. In cases where the mass has been identified to be benign, but the organ has been opened, removal of splenic tissue may be performed. With SGF, there may be no need for surgery. However, diagnosis and surgical treatment by a diagnostic laparoscopy are suggested. Laparoscopy is a safe and reliable approach that is highly accurate in the diagnosis and treatment of nonpalpable testis.
